# FRAILTY AND COGNITIVE IMPAIRMENT: RESULTS FROM A DIVERSE SAMPLE OF OLDER PERSONS RECEIVING HEMODIALYSIS

**DOI:** 10.1093/geroni/igad104.3359

**Published:** 2023-12-21

**Authors:** Ana Alfaro, Rachel Bryan, Swathi Balantrapu, Christine Liu

**Affiliations:** Stanford School of Medicine, Stanford, California, United States; Stanford School of Medicine, Stanford, California, United States; Stanford School of Medicine, Stanford, California, United States; Stanford School of Medicine, Stanford, California, United States

## Abstract

Persons who are receiving hemodialysis are at high risk for frailty and cognitive impairment. However, little is known about the overlap of these conditions in a diverse older hemodialysis population. Our goal is to examine frailty and cognition in a diverse sample of older adults receiving hemodialysis. Data was collected as part of a cross-sectional observational study examining mentation, mood, and mobility. Frailty was identified using the Study of Osteoporotic Fractures Index and cognition was assessed with the Montreal Cognitive Assessment (MoCA) (cut off score ≤ 26). Blind MoCAs were administered (cut off score ≤ 22) when appropriate. 35 participants (mean age = 73.9 ± 10.7) enrolled. 60% were males and 20% were Spanish-speaking. 20% identified as Asian, 23% as Black, and 23% as Hispanic/ Latino. Of note, four blind MoCAs in Spanish were administered. 83% had abnormal MoCA scores. 86% were frail or pre-frail. The majority of the sample (69%) had concurrent cognitive impairment and frailty or pre-frailty. In this diverse sample of older adults receiving hemodialysis, we found that both frailty and cognitive impairment are extremely common, with a large portion of the sample exhibiting both. This suggests that interventions targeting cognitive impairment or frailty should account for the frequent overlap of both conditions, particularly among historically underserved populations.

